# Supplementary material for: Prediction Model of Hemorrhage Transformation in Patient with Acute Ischemic Stroke Based on Multiparametric MRI Radiomics and Machine Learning
Source: Brain Sci. 2022 Jun 29;12(7):858. doi: 10.3390/brainsci12070858 (PMC9313447; doi:10.3390/brainsci12070858)
Supplement: Supplementary file 1 [file brainsci-12-00858-s001.zip › brainsci-1779440-supplementary.pdf]

brain sciences

MDPI

Article

Prediction Model of Hemorrhage Transformation in Patient with Acute Ischemic Stroke Based on Multiparametric MRI Radiomics and Machine Learning

Yucong Meng [1,2,†], Haoran Wang [1,2,†], Chuanfu Wu [1,2,†], Xiaoyu Liu [1,2], Linhao Qu [1,2] and Yonghong Shi [1,2,*]

[1] Digital Medical Research Center, School of Basic Medical Sciences, Fudan University, Shanghai 200032, China; ycmeng21@m.fudan.edu.cn (Y.M.); hrwang20@fudan.edu.cn (H.W.); cfwu14@fudan.edu.cn (C.W.); liuxiaoyu21@m.fudan.edu.cn (X.L.); lhqu20@fudan.edu.cn (L.Q.)

[2] Shanghai Key Laboratory of Medical Imaging Computing and Computer Assisted Intervention, Shanghai 200032, China

* Correspondence: yonghong.shi@fudan.edu.cn

† These authors contributed equally to this work.

Table S1. Classification accuracy of models with different number of estimators (n_estimators).

n_estimators	AUC	ACC	SEN	SPEC	F1-Score
10	0.804 ± 0.015	0.803 ± 0.021	0.600 ± 0.163	0.863 ± 0.055	0.571 ± 0.070
20	0.839 ± 0.022	0.818 ± 0.000	0.733 ± 0.094	0.706 ± 0.220	0.843 ± 0.028
30	0.861 ± 0.003	0.803 ± 0.021	0.600 ± 0.000	0.863 ± 0.028	0.582 ± 0.026
40	0.855 ± 0.006	0.833 ± 0.043	0.733 ± 0.094	0.863 ± 0.028	0.667 ± 0.086
50	0.859 ± 0.010	0.833 ± 0.043	0.733 ± 0.094	0.863 ± 0.028	0.667 ± 0.086
60	0.867 ± 0.011	0.833 ± 0.021	0.600 ± 0.000	0.902 ± 0.028	0.622 ± 0.031
70	0.867 ± 0.006	0.864 ± 0.037	0.733 ± 0.094	0.902 ± 0.028	0.709 ± 0.083
12	0.835 ± 0.017	0.758 ± 0.021	0.800 ± 0.000	0.745 ± 0.028	0.601 ± 0.021
80	0.867 ± 0.011	0.864 ± 0.037	0.733 ± 0.094	0.902 ± 0.028	0.709 ± 0.083
90	0.867 ± 0.020	0.833 ± 0.043	0.667 ± 0.094	0.882 ± 0.048	0.646 ± 0.076
100	0.871 ± 0.019	0.848 ± 0.021	0.733 ± 0.094	0.882 ± 0.048	0.687 ± 0.029
200	0.855 ± 0.020	0.833 ± 0.021	0.667 ± 0.094	0.882 ± 0.048	0.644 ± 0.031
300	0.855 ± 0.015	0.833 ± 0.043	0.733 ± 0.094	0.863 ± 0.028	0.667 ± 0.086
400	0.863 ± 0.006	0.833 ± 0.043	0.733 ± 0.094	0.863 ± 0.028	0.667 ± 0.086
500	0.863 ± 0.011	0.818 ± 0.000	0.667 ± 0.094	0.863 ± 0.028	0.622 ± 0.031
600	0.851 ± 0.011	0.833 ± 0.043	0.733 ± 0.094	0.863 ± 0.028	0.667 ± 0.086
700	0.855 ± 0.006	0.818 ± 0.037	0.667 ± 0.094	0.863 ± 0.028	0.624 ± 0.076
800	0.855 ± 0.015	0.833 ± 0.021	0.733 ± 0.094	0.863 ± 0.028	0.665 ± 0.052
900	0.855 ± 0.006	0.818 ± 0.000	0.660 ± 0.094	0.863 ± 0.028	0.622 ± 0.031
1000	0.855 ± 0.006	0.818 ± 0.000	0.667 ± 0.094	0.863 ± 0.028	0.622 ± 0.031

Table S2. Classification accuracy of models with different subtree's max_depth.

Max_depth	AUC	ACC	SEN	SPEC	F1 Score
1	0.816 ± 0.022	0.742 ± 0.021	0.000 ± 0.000	0.961 ± 0.028	0.000 ± 0.000
2	0.855 ± 0.006	0.803 ± 0.057	0.733 ± 0.189	0.824 ± 0.127	0.631 ± 0.027
3	0.871 ± 0.000	0.803 ± 0.021	0.600 ± 0.000	0.863 ± 0.028	0.582 ± 0.026
4	0.867 ± 0.015	0.833 ± 0.021	0.667 ± 0.094	0.882 ± 0.048	0.644 ± 0.031
5	0.871 ± 0.019	0.848 ± 0.021	0.733 ± 0.094	0.882 ± 0.048	0.687 ± 0.029
6	0.869 ± 0.017	0.818 ± 0.037	0.600 ± 0.000	0.882 ± 0.048	0.604 ± 0.050
7	0.869 ± 0.017	0.818 ± 0.037	0.600 ± 0.000	0.882 ± 0.048	0.604 ± 0.050
8	0.869 ± 0.017	0.818 ± 0.037	0.600 ± 0.000	0.882 ± 0.048	0.604 ± 0.050
9	0.869 ± 0.017	0.818 ± 0.037	0.600 ± 0.000	0.882 ± 0.048	0.604 ± 0.050
10	0.869 ± 0.017	0.818 ± 0.037	0.600 ± 0.000	0.882 ± 0.048	0.604 ± 0.050
None	0.869 ± 0.017	0.818 ± 0.037	0.600 ± 0.000	0.882 ± 0.048	0.604 ± 0.050
